# Correlation of Indoxyl Sulfate to Hearing Impairment in Chronic Kidney Disease Patients

**DOI:** 10.22038/ijorl.2025.77733.3633

**Published:** 2025

**Authors:** Fadillah-Akbar Sanjani, Tengku-Siti-Hajar Haryuna, Farhat Farhat, Syafrizal Nasution, Juliandi Harahap, Yuliani M Lubis, Harry-Agustaf Asroel, Khalisanni Khalid

**Affiliations:** 1 *Department of Otorhinolaryngology - Head and Neck Surgery, Faculty of Medicine, Universitas Sumatera Utara, Jl. Dr. Mansur No. 5, Medan, 20155, Indonesia.*; 2 *Department of Internal Medicine, Faculty of Medicine, Universitas Sumatera Utara, Jl. Dr. Mansur No. 5, Medan 20155, Indonesia.*; 3 *Department of Community Medicine, Faculty of Medicine, Universitas Sumatera, Utara, Jl. Dr. Mansur No. 5, Medan, 20155, Indonesia.*; 4 *Malaysian Agricultural Research and Development Institute (MARDI), MARDI Headquarters, Persiaran MARDI-UPM, 43400 Serdang, Selangor, Malaysia.*

**Keywords:** Chronic kidney disease, Indoxyl sulfate, Hearing loss, Pure tone audiometry, OAE

## Abstract

**Introduction:**

Chronic Kidney Disease (CKD) is considered a public health issue because its frequency is increasing in adults. When a person experiences renal failure, one of the most researched solutes that builds up in plasma is indoxyl sulfate. This toxin can attach to proteins, and it is a byproduct of the tryptophan metabolism in the diet, which provides pro-oxidative and pro-inflammatory activity. In CKD, the redox imbalance associated with oxidative stress is associated with pathophysiological issues brought on by the buildup of uremic toxins. The cochlea is highly susceptible to oxidative stress, which consequently causes permanent cochlear degeneration. To better understand the connection between Indoxyl sulfate levels and hearing loss in CKD patients, we examined the results of pure tone audiometry and OAE examinations.

**Materials and Methods:**

This research was conducted on 27 people with stage 5 CKD who had their blood plasma levels of indoxyl sulfate measured before having their hearing ability assessed by OAE and pure tone audiometry. Next, a correlation test was carried out between the results of Indoxyl sulfate levels and the results of hearing function tests in CKD patients.

**Results:**

The indoxyl sulfate value and degree of auditory impairment had a strong positive correlation, according to the Spearman correlation test. (r = 0.881; p = 0.001) and an inverse relationship between the Indoxyl sulfate value and SNR (r = -0.761; p = 0.001).

**Conclusion:**

CKD patients have impaired hearing, which is correlated with the amount of uremic toxin Indoxyl Sulfate that has accumulated.

## Introduction

It took over eight decades for the connection between CKD and deafness to be documented. CKD has become an important issue in public health because of how often it affects people. Several studies have shown a possible link between the ears and the kidneys (1).

Sensory Neural Hearing Loss (SNHL) is a decrease in hearing acuity caused by lesions in the cochlea and/or cochlear nerve. It can be unilateral or bilateral, permanent or reversible. SNHL can occur in cases of CKD undergoing hemodialysis. SNHL in CKD sufferers undergoing hemodialysis occurs at various frequencies (2). 

Hemodialysis is a blood cleansing procedure through an artificial kidney (dialyzer) and is assisted by a machine. A semipermeable synthetic membrane replaces the glomerulus and renal tubules and works as a filter for kidneys whose function is impaired. Hemodialysis is performed to dilute the blood and eliminate nitrogen compounds that are harmful to the body (3). In CKD, nephron damage occurs so that renal excretory function decreases and there is an accumulation of toxins, electrolyte disturbances, metabolic acidosis, and calcium metabolism disorders, which can affect the cochlea and interfere with hearing function (3). SNHL in CKD sufferers undergoing hemodialysis occurs at various frequencies. CKD can cause the toxic condition of uremia, which can cause abnormalities at high frequencies (>2000 Hz). 

This situation usually does not cause complaints of poor hearing because it does not affect the speaking frequency (500 Hz- 2000 Hz). Stavroulaki et al. 15 out of 28 patients (54%) who were having hemodialysis were found to have high-frequency hearing loss (>2,000 Hz). They also found that the length of time a patient spent on hemodialysis was associated with the likelihood of hearing loss; specifically, 30% of individuals undergoing hemodialysis for 18 months or longer had hearing loss, and 67% of individuals undergoing hemodialysis for over 18 months also had this problem (3,4). 

One of the most researched solutes that builds up in plasma in patients with renal failure is indoxyl sulfate. People with renal illness had high amounts of it in their blood when it was initially isolated in 1911 by Obermayer and Popper. At first, its function as a "putrefaction" byproduct of colonic microbial metabolism piqued his clinical interest outside of renal illness. Research conducted in the 1950s examined the potential associations between indoxyl sulfate and several medical issues, especially gastrointestinal and mental illnesses. Since the kidney is known to be the primary organ responsible for clearing indoxyl sulfate, attention has turned to its possible involvement in renal disease. Since then, other investigations have evaluated the role of indoxyl sulfate in kidney disease side effects (5).

When ROS levels rise and antioxidant capacity falls, a redox imbalance has occurred, leading to oxidative stress. Oxidative stress disrupts normal physiological function by damaging cellular carbohydrates, lipids, proteins, and nucleic acids. The development of atherosclerotic lesions, a risk factor for cardiovascular disease, is known to be triggered by oxidative stress. In CKD, the buildup of uremic toxins is associated with pathological consequences through oxidative stress-induced redox imbalance. Normally eliminated by the kidneys, uremic toxins build up in the blood as CKD progresses. Dialysis has a hard time eliminating protein-bound uremic toxins, although their molecular weight is around 500 daltons. Their ability to form bonds with proteins is the reason for this. The majority of these uremic toxins are attached to proteins and consist of phenolic compounds derived from tyrosine and phenylalanine, and indolic compounds derived from tryptophan, as indoxyl sulphate, which are created by gut bacterial fermentation. Interest in CKD has lately increased due to the role that uremic toxins play in redox imbalance. Uremic toxin-related redox abnormalities are widespread in CKD patients, and they frequently exacerbate CKD problems over time. Indoxyl sulfate has been demonstrated to have pro-oxidant effects on a variety of exposed tissues. Thus, investigating the pro-oxidant role of Indoxyl sulfate is essential for CKD-related complications (6). Because its mechanosensory hair cells undergo such high metabolic demands in reaction to auditory inputs, the cochlea is especially vulnerable to damaging free radicals. Under normal physiological settings, the endogenous antioxidant systems of hair cells normally break down ROS produced in the mitochondria of hair cells. Peroxidation of lipids, depolymerization of polysaccharides, disruption of nucleic acids, oxidation of sulfhydryl groups, and inactivation of enzymes are some of the genetic and cellular alterations that lead to cellular malfunction and, ultimately, irreversible cochlear degeneration caused by elevated ROS concentrations (7).

Prior research in individuals with chronic renal illness has not examined the link between indoxyl sulphate concentrations and diminished auditory function. In light of this, studies investigating the link between elevated indoxyl sulfate and diminished auditory function in CKD patients are welcome.

Based on the preceding description, we hypothesize that elevated levels of indoxyl sulfate are linked to hearing impairment caused by uremic syndrome.

## Materials and Methods

To ascertain the relationship between serum levels of indoxyl sulfate and hearing loss in sufferers with CKD, this investigation is a cross-sectional study using observational data. Purposive sampling is used as a sample selection technique. All subjects in the existing population who meet the inclusion criteria are included in the research sample until the entire sample size is met. This research was carried out in a sound treated room with hearing loss examinations using pure tone audiometry, and OAE at Haji Adam Malik Hospital, Medan. Examination of Indoxyl sulfate levels was carried out at the Integrated Laboratory of the Faculty of Medicine, University of North Sumatra. Pure tone audiometry and OAE examination using a device with the GSI brand (Grasson-Stadler Corti, USA). Examination of Indoxyl sulfate levels using the Human Indoxyl Sulfate ELISA Kit, Bioassay brand. This research was conducted from October to December 2023.

This research categorized CKD according to the extent of renal failure, which was evaluated by calculating the GFR derived from blood creatinine. The following frequencies were used for pure tone audiometry: 8000 Hz, 4000 Hz, 2000 Hz, 1000 Hz, 500 Hz, 250 Hz. We recorded the results of the audiometry tests and categorized the patients based on the WHO guidelines for pure tone audiograms. Every frequency has its own NF and SNR number on DPOAE, which was calculated from the discrepancy in amplified DP. To pass, the SNR value had to be more than 6, and to refer, it had to be less than 6. 

### Sample

An example the participants in the study were adults (16–60 years old) with a history of kidney disease (ranging from less than 5 years to more than 5 years), who were undergoing routine hemodialysis at the Hemodialysis Room at Haji Adam Malik Hospital in Medan. A Nephrology Consultant Internal Medicine Specialist had previously diagnosed CKD. The participant’s normal ENT examination results were also taken into consideration, and they were willing to take part in the research. 

Patients with disorders in the findings of standard ENT examinations were excluded from this investigation, such as infection, otitis media, head trauma in the last 2 weeks, patients who refused to participate in the study, patients in poor general health, and patients who were uncooperative during the test. Based on the proportion formula, the sample size is 27 patients.

### Procedure

To ensure that all procedures carried out in this research are ethical, before the research is carried out, the proposal must first be submitted to the Research Ethics Commission of the University of North Sumatra to obtain an assessment and approval of ethical feasibility with the number: 1237/KEP/USU/2022.

CKD sufferers are being treated in the hemodialysis unit at RSUP. H. Adam Malik Medan, who met the inclusion criteria, was given an explanation to ask for his availability to be involved in the research, then asked to fill out informed consent. Patients who were willing to be involved in the study recorded their name, gender, age, and duration of hemodialysis, with the results of routine ENT examinations within normal limits. Hearing function was examined using pure tone audiometry and OAE. The levels of indoxyl sulfate were then measured, and a 3 ml blood sample was obtained right before the patient received hemodialysis. 

Blood samples were taken from the cubital vein of the research participant's arm by a health worker, then put into a blood tube containing EDTA, then the blood plasma was stored in a cooler box or left for 2 hours at room temperature and could be left overnight at 2^o^C-8^o^C before being centrifuged in the USU Faculty of Medicine Integrated Laboratory. Examination of indoxyl sulfate levels using the ELISA/ Enzyme-Linked Immunosorbent Assay method.

### Statistical analysis

The data obtained is presented in tabular form so that a descriptive picture of all the variables studied can be seen. To see the correlation between the independent variable, namely Indoxyl sulfate levels, and the dependent variable, when analysing the data, the Pearson correlation test was used to see whether the data followed a normal distribution. Whether or not the Spearman correlation test was used in the absence of normal distribution. Both a correlation coefficient and a significance value are generated by the correlation test; a p-value less than 0.05 is deemed important using SPSS v.23. 

## Results

### Univariate analysis

From Table 1, it was found that the research subjects with the largest gender were men at 51.9%. The biggest demographic by age is the 46–60-year age group at 59.3%. Most of the samples suffered from CKD for ≥5 years, 51.9%.

**Table 1:** Demographic Characteristics of Research Subjects

**Demographic Characteristics**	**n = 27**
Gender,	**n (%)**
Man	**14** ** (51,9)**
Woman	**13 (48,1)**
**Age, years**	
**<25 years**	**0 (0)**
**25-35 years**	**5 (18,5)**
**36-** **4** **5 years**	**6 (22,2)**
**45** **-** **60** ** years**	**16 (59,3)**
Duration of CKD	
<5 years	**13 (48,1)**
≥5 years	**14 (51,9)**

### Univariate analysis

From Table 2, it is known that the majority of subjects experienced moderate hearing loss, 14 people (51.9%), followed by respondents with mild hearing loss, 9 people (33.3%), and respondents with moderate-severe hearing loss, 4 people (14.8%). Most of the subjects had SNR passes, namely 16 people (59.3%), and SNR refer as many as 11 people (40.7%).

Hearing Loss Assessment

**Table 2:** Frequency Distribution of Subjects Based on Degree of Hearing Loss and SNR

**Hearing Loss Assessment**	**n = 27**
Degree of hearing loss	
Mild	**9 (33,3)**
Moderate	**14 (51,9)**
Moderately Severe	**4 (14,8)**
**SNR**	
***Pass***	**16 (59,3)**
***Refer***	**11 (40,7)**

### Descriptive analysis

From Table 3, it is known that the average value of indoxyl sulfate is 78.62, with an SD of 79.59. The median value was 42.5, with a minimum of 12.9 and a maximum of 245.0.

**Table 3:** Average values ​​of Indoxyl sulfate

**Variable**	**Mean±SD**	**Median (Min-Max)**
*Indoxyl sulfate*	78,62 ± 79,59	42,5 (12,9 – 245,0)

**Table 4:** Correlation of the Length of Suffering from CKD on Degree of Hearing Loss and SNR

**Variable **	**Duration of CKD**				**Amount **		**p-value**
	**<5 years**		**>5 years**				
	**f**	**%**	**F**	**%**	**f**	**%**	
Degree of hearing loss							
**Mild**	9	33,3	0	0,0	9	33,3	0,001
**Moderat** **e**	4	14,8	10	37,0	14	51,9	
**Moderately Severe**	0	0,0	4	14,8	4	14,8	
Amount	13	48,1	14	51,9	27	100,0	
SNR							0,001
Pass	13	48,1	3	11,1	16	59,3	
Refer	0	0,0	11	40,7	11	40,7	
Amount	13	48,1	14	51,9	27	100,0	

### Bivariate analysis

From Table 4, it is known that based on the duration of suffering from CKD, the majority of respondents with a duration of suffering from CKD <5 years experienced mild disorders in as many as 9 people (33.3%) and SNR pass as many as 13 people (48.1%) and a duration of suffering from CKD ≥ 5 years, 4 people (14.8%) experienced moderate to severe hearing loss and 11 people (40.7%) had SNR refer. The results of statistical analysis show a p-value of 0.001, meaning that a link exists between the duration of CKD and the degree of hearing loss and OAE.

**Table 5:** Correlation of Indoxyl sulfate level with Degree of hearing loss and SNR

**Variable**		**r-value**	**p-value**
Indoxyl Sulfate	Degree of hearing loss	0,881	0,001*
	SNR	-0,761	0,001*

*Spearman correlation

The normality test results showed that the degree of hearing loss, SNR, and indoxyl sulfate values ​​were not normally distributed, namely 0.200 (p>0.05). From Table 5, it is known that indoxyl sulphate had a very significant positive association with the degree of auditory impairment (r = 0.881; p = 0.001), on top of the fact that indoxyl sulphate and SNR are strongly inversely related. (r = -0.761; p = 0.001).

## Discussion

This research was conducted on patients with CKD stage 5. In this investigation, the majority of subjects aged 46-60 years were 16 people (59.3%), subjects aged 36-45 years were 6 people (22.2%), and subjects aged 25-35 years were 5 people (18.5%). This result follows the results of the 11th Report of the Indonesian Renal Registry (2018), which states that patients aged 45 to 64 still make up the greatest percentage of the patient population.^8^ This is supported by research conducted by Aisara (2018), which discovered that the majority of hemodialysis individuals at RSUP Dr. M. Djamil Padang are between the ages of 40 and 60. It is natural for individuals to see a decline in kidney function as they become older. Renal Blood Flow (RBF) and Glomerular Filtration Rate (GFR) both decline with age. Beginning at age 40, there is a gradual decline of around 8 ml/minute/1.73m2 every decade (9).

From the results of research from the 11th Report of the Indonesian Renal Registry (2018), according to reports, men in Indonesia are more likely than women to have CKD.^8^ In line with research conducted by Aisara (2018), a comparison of CKD undergoing hemodialysis at RSUP Dr. M. Djamil Padang based on male and female gender is three to two. In this investigation, similar results were obtained where the incidence of CKD in men was higher than in women, with 14 men (51.9%) and 13 women (48.1%). These results may be related to the incidence of diseases that cause CKD, such as kidney stones, which also occur more often in men. Another study found that the frequency of kidney stones among males and females was 10.6% and 7.1% (9). Sexual dimorphism in the development of CKD is affected by several potential dangers, including hypertension, hyperglycemia, dyslipidemia, BMI, albuminuria, and others, such as lifestyle, kidney structure, and sex hormones (10). Basically, this disease can attack anyone. Therefore, both men and women are encouraged to adopt a healthy lifestyle.

In this investigation, there were 14 subjects with moderate hearing loss (51.9%), 9 people with mild hearing loss (33.3%), and 4 people with moderately severe hearing loss. (14.8%). For SNR, it was found that 16 subjects with pass examination results (59.3%) and 11 subjects (40.7%) refer. Research conducted by Ulfa (2016) found that the incidence of Sensorineural Hearing Loss was 8 out of 26 subjects in the HD group (p=0.004), consisting of mild SNHL in 7 subjects and moderate SNHL in one subject (2). Meanwhile, from the research conducted by Pushpa (2021), out of a total of 115 correspondents, 40% experienced SNHL, of which 13% experienced very mild, 8.7% moderate, 0.9% very severe, 13% mild, 0.9% severe, and 3.5% moderately severe (11). 

Purnami (2022) discovered that out of 25 patients (83.3% of the total), DPOAE exhibited refer findings, while 5 patients showed pass results (12). In addition to accurately evaluating hearing function, a pure tone audiometry test may provide information specific to frequencies. As far as anybody knows, cochlear dysfunction may cause distortionary otoacoustic emission products (DPOAEs). Evaluating and providing information on cochlear function in sufferers with CKD may be done using either DPOAE or pure tone audiometry (13).

Sensorineural hearing loss was prevalent in CKD patients in recent investigations. The CKD group had reduced cochlear activity. When compared with controls, all CKD patients had significantly reduced SNR values. Even when their hearing thresholds are adequate, people with CKD have a malfunction in their outer hair cells. This might suggest that malfunctioning of the outer hair cells is the first manifestation of inner ear abnormalities caused by CKD in DPOAE (14). Research conducted by Reddy (2016) examined 200 sufferers with CKD. The results demonstrated a strong relationship during the time frame of CKD and the threshold for auditory impairment (15). Consistent with the investigation, 21 out of 29 patients (72.4% of the total) with CKD lasting longer than 5 years had hearing loss (Pushpa, 2021). Just 22 instances (37.9%) out of 58 cases with a period of 1 to 4 years had hearing loss, while out of 28 cases with a duration of less than 1 year, just 3 cases (10.7%) did the same. These are corroborated by the findings of Somashekara (2015), who found a correlation between hearing loss and CKD, prolonged hemodialysis treatment, and electrolyte imbalances (16). In this research, 14 participants (51.9% of the total) had CKD for more than five years, whereas 13 participants (48.1% of the total) had CKD for less than five years. This investigation's findings that an association between CKD duration, hearing loss severity, and SNR is in line with those of Reddy, Pushpa, and Somashekara. The research indicated that in terms of degree of auditory impairment and SNR, CKD patients with a length of more than 5 years of suffering had a higher incidence of hearing loss compared to CKD patients with a period of less than 5 years (17).

Serum Indoxyl sulfate levels are positively correlated with the length of time a patient has been on hemodialysis for CKD, according to studies done by Pebriarti (2018). 17 There was a very significant positive association between IS and degree of hearing loss, as shown by the study's findings: a correlation coefficient of 0.881 and a p-value of 0.001 (p<0.05). A high negative connection between IS and SNR was shown by the r-value of -0.761 and the p-value of 0.001 (p<0.05) for IS on SNR. The results of this research show that CKD patients had higher-than-average plasma indoxyl sulfate levels, with a mean of 78.62 µg/ml and a SD of 79.59 µg/ml. The range of plasma indoxyl sulfate levels was 12.9 µg/ml to 245 µg/ml. Indoxyl sulfate levels were found to be higher in individuals with CKD, according to a study published by Pebriarti (2018). With an SD of 23.205 µg/mL, the average serum Indoxyl sulfate level was 37.062 µg/mL. The serum Indoxyl sulfate levels ranged from 11.693 µg/mL at the lowest end to 166.365 µg/mL at the highest end. The indoxyl sulfate level is the measure of the quantity of indoxyl sulfate in the blood, which includes serum and plasma. There is currently no universally accepted reference value for indoxyl sulfate levels. Several investigations, however, found that indoxyl sulphate concentrations in physiologically fit people were almost nonexistent (18).

The uremic toxin accumulation, Indoxyl sulfate, is toxic to the designated organ in individuals suffering from chronic renal failure. In CKD, indoxyl sulfate promotes ROS formation, which in turn disrupts glomerular filtration by directly cytotoxically impacting mesangial cells. Oxidative stress occurs when there is a redox imbalance, defined as an excess of ROS/RNS and a deficiency of antioxidant capability. Damage from ROS/RNS does not affect all cochlear cell types equally. When it comes to free radical damage, the outer hair cells are the most vulnerable, but the supporting cells are much more likely to survive (19).

In this investigation, a strong relationship was found between Indoxyl sulfate levels, degree of hearing loss, and SNR. No studies have been identified that aim to establish a connection between Indoxyl sulphate levels and auditory function as of yet. We acknowledge that there are still limitations to this investigation. Constraints on this investigation were that the sample selection criteria did not exclude patients with diabetes and hypertension. More investigation is needed to establish the consequences of antioxidants and other therapies on Indoxyl sulfate levels and hearing function, and to evaluate the effects of additional uremic toxins known to induce hearing loss. 

## Conclusion

Patients with CKD tend to experience sensorineural hearing loss. The duration of suffering from CKD and Indoxyl sulfate levels have a significant correlation with hearing function. Any procedure worth its salt should include regular hearing tests to manage sufferers with CKD, for the prompt identification of auditory impairment so that immediate action can be undertaken.
